# Discovery of microRNAs in *Pyrus* stigma exudates opens new research avenues in Horticulture

**DOI:** 10.1093/pnasnexus/pgad332

**Published:** 2023-10-12

**Authors:** Vivek Ambastha, Yuval Nevo, Ifat Matityhu, David Honys, Yehoram Leshem

**Affiliations:** Department of Plant Sciences, MIGAL—Galilee Research Institute, Kiryat-Shmona 11016, Israel; Info-CORE, Bioinformatics Unit of the I-CORE, The Hebrew University of Jerusalem, Jerusalem 9112001, Israel; Department of Plant Sciences, MIGAL—Galilee Research Institute, Kiryat-Shmona 11016, Israel; Laboratory of Pollen Biology, Institute of Experimental Botany of the Czech Academy of Sciences, Rozvojová 263, 165 02 Prague 6, Czech Republic; Department of Plant Sciences, MIGAL—Galilee Research Institute, Kiryat-Shmona 11016, Israel; Faculty of Sciences and Technology, Tel-Hai College, Upper Galilee 1220800, Israel

**Keywords:** stigma exudates, extracellular RNA (exRNAs), microRNA (miRs), pear species

## Abstract

In many plant species, flower stigma secretions are important in early stages of sexual reproduction. Previous chemical analysis and proteomic characterization of these exudates provided insights into their biological function. Nevertheless, the presence of nucleic acids in the stigma exudates has not been previously reported. Here, we studied the stigma exudates of *Pyrus communis*, *Pyrus pyrifolia*, and *Pyrus syriaca* and showed them to harbor extracellular RNAs of various sizes. RNA sequencing revealed, for the first time, the presence of known Rosaceae mature microRNAs (miRs), also abundant in the stigma source tissue. Predicted targets of the exudate miRs in the *Arabidopsis thaliana* genome include genes involved in various biological processes. Several of these genes are pollen transcribed, suggesting possible involvement of exudate miRs in transcriptional regulation of the pollen. Moreover, extracellular miRs can potentially act across kingdoms and target genes of stigma interacting organisms/microorganisms, thus opening novel applicative avenues in Horticulture.

## Introduction

The stigma is a glandular female organ, which receives the male gametophyte during the beginning of sexual reproduction in flowering plants [1]. These plants can be divided into species possessing either a “dry” or “wet” stigma, which depends on absence or presence of liquid excretions at the stigma surface, or a “semi-dry” stigma, with features of both dry and wet stigmas ([2, 3]. These exudates can be lipidic or aqueous and carbohydrate-rich. Both secretion types also contain proteins and additional components such as Ca^2+^ and other ions, amino acids, phenols, and reactive oxygen species [1, 4]. The stigma exudates play a key role in pollen capture, pollen adhesion, hydration, germination, sustenance of early tube growth, and the capture of various microorganisms [1, 4]. New insights into the exudate roles were gained through a proteomic study, which identified in the stigma secretome many unique proteins which were reported to be involved in multiple biological processes [5]. Therefore, this extracellular medium is apparently biochemically active, and possibly functional in wider biological contexts than described above [4].

Over the last decade, RNA biology has acquired novel insights with the findings that RNA is found extracellularly and conveys information between cells, organisms, and species. Since then, the new field of extracellular RNA (exRNAs) has been extensively studied in mammalians systems, where exRNAs (including extracellular microRNAs [miRs]) were found to be associated with various diseases [6]. However, in plants, this field has been studied to a much lesser extent and our understandings of plant exRNA modes of action is still very limited [7].

Until now, to our knowledge, the presence of miRs, small noncoding RNAs that regulate transcription, have not been previously reported in stigma exudates. Previous partial characterization of the stigma exudate of *Pyrus communis* showed that it comprised several free sugars and free amino acids, however, nucleic acids were not discovered in that work [8].

Due to the great regulatory impact of miRs on a wide range of plant processes [9], and their tiny molecular size that would allow easy secretion from cells, we explored and discovered in three *Pyrus* species, *P. communis*, *Pyrus pyrifolia*, and *Pyrus syriaca*, the presence of miRs in stigma exudates.

## Results and discussion

**Fig. 1.**
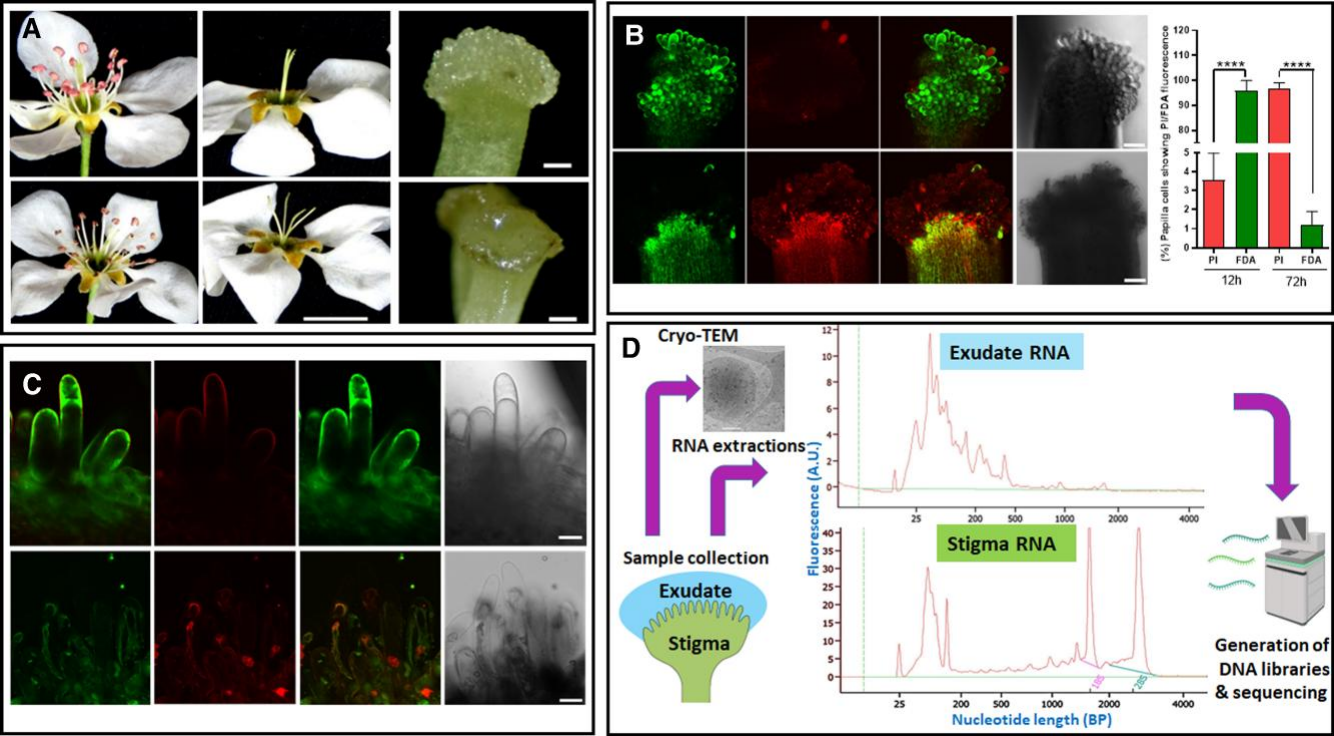
Stigma viability during blooming progression and features of exudate RNA in *Pyrus communis*. A) Stigma and anther positions 12 h (upper row) and 72 h (lower row) after anthesis. On the right-hand side are stereoscope-magnified images of the stigma at the mentioned time points. B) Confocal microscopy images of the stigma stained with FDA and PI 12 HAA (upper row) and 72 HAA (lower row). The green fluorescence (FDA) indicates viable cells, while the nucleus-localized red fluorescence (PI) indicates dead cells. The images represent maximum intensity projection along the Z-axis, where each image stack covers 3 µm. The histogram on the right side represents the percentage viability of stigma tissue 12 and 72 HAA. Quantification was performed for n = 9 stigmatic lobes. Asterisks indicate statistical significance at *P* < 0.0001 after applying the Mann–Whitney nonparametric test. C) High magnification confocal images of papilla cells 12 HAA (upper row) and 72 HAA (lower row), stained with FDA (green fluorescence) and PI (red fluorescence). The images present a single layer of 2-µm thick sections (BF, bright field). Scale bars in A = 1 cm for the flowers and 100 µm for individual stigma; while in B = 50 µm and in C = 20 µm. D) Schematic diagram of RNA and extracellular vesicle (EV) isolation from stigma and exudates. The electropherograms on the right show characteristics of RNA extracted from exudates (top) and stigma (bottom). Note that two sharp peaks corresponding to 18S and 28S rRNA, respectively, are visible in the stigma but are completely absent in the exudates. A.U. and BP are arbitrary units and base pairs, respectively. Details of the exudate Cryo-TEM analysis of the EV depicted at the top left of D are provided in supporting dataset S5. The sequencer image was adopted from BioRender (BioRender.com).

The flower of *P. communis* is hermaphroditic with a pentacarpellar syncarpous gynecium. Upon anthesis, the stigma is highly receptive [10], its surface is already wet, and its lobes are clustered together facing upwards. As blooming progresses, the stigma receptivity drops and its lobes bifurcate sideward [10] (Fig. 1A). To determine whether the browning of the stigma edges, observed during late blooming, indicates senescence, we determined in non-pollinated stigmas, the papilla cell viability at 12 and 72 hours after anthesis (HAA). Using confocal fluorescent microscopy, we examined the signal of the live/dead stains Fluorescein diacetate (FDA) and Propidium Iodide (PI), respectively, which were applied simultaneously. At 12 HAA, strong FDA cytoplasmic signal was observed while the PI signal intensity was low and distributed around the cell periphery. The stigmatic papilla cells at this stage were thus intact and highly viable. However, at 72 HAA, this pattern was reversed: A distinct PI signal was observed in the papilla cell nuclei while the FDA signal was almost absent, indicating that the papilla cell viability had dropped sharply at 72 HAA (Figs. 1B and C). Similar viability staining patterns were observed in stigmas of *P. syriaca* and *P. pyrifolia*.

To avoid characterizing exudates of dead/dying cells, which may arise from membrane leakage rather than active secretion, we studied the stigmas at 12 HAA, for which papilla cell viability and integrity had been confirmed. The exudates were sampled in a nondestructive manner from non-pollinated stigmas. As a reference tissue, non-pollinated stigmas were also sampled. We then extracted RNA from the collected samples and evaluated their concentration electrophoretically. As expected, the exudate's total RNA yield was much lower than the yield obtained from the stigma. Moreover, the exudate samples were free of ribosomal RNA (rRNA), as opposed to the stigma samples in which rRNA (mainly the 18S and 28S sub-units) was highly abundant (Fig. 1D). The exudate RNA features of *P. pyrifolia* and *P. syriaca* were similar. The lack of rRNA in the exudate implies that at 12 HAA the stigma exudates are not cytoplasmic spillover of lysed stigmatic cells (which would allow nonselective passage of large molecules such as the rRNA 18S and 28S sub-units), but rather, the secretion of non-rRNA from stigmas of intact cells. Further examination of the exudate RNA electropherograms indicated the presence of small RNAs of various sizes (shorter than 200 nucleotides in length) (Fig. 1D).

**Fig. 2.**
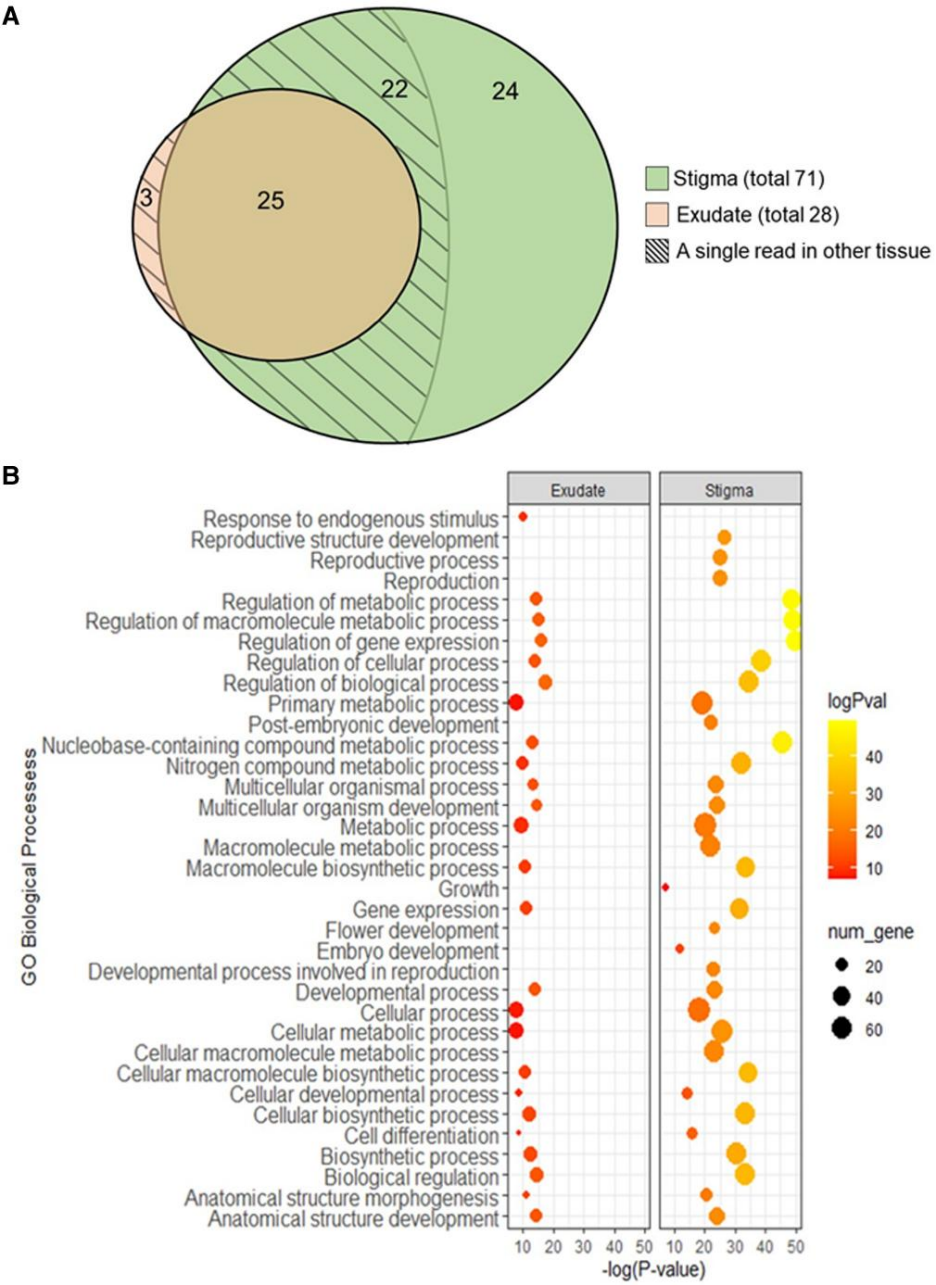
Mature Rosaceae miRs identified in stigma and stigma exudates of several *Pyrus* species and functional categorization of their putative targets in the Arabidopsis genome. A) Venn diagram of identified mature miRs from stigma (green) and stigma exudates (orange) of *Pyrus communis*, *Pyrus pyrifolia*, and *Pyrus syriaca*. Shown are the number of miRs common to both tissues (25) and stigma-specific miRs (24). Special areas, marked with diagonal lines, are for miRs with exactly one read in the other tissue, making them neither common to both nor tissue-specific. Full details of the common and tissue-specific miRs are given in supporting dataset S1. B) Dot plot representation of enriched Gene Ontology (GO) terms for biological processes (BP) of 120 and 47 Arabidopsis genes that are predicted targets of pear stigma and exudate miRs presented in A, respectively. Y-axis indicates the GO term, and X-axis shows the count of genes per GO term. Color gradient indicates negative log of *P*-value. Full details are presented in supporting datasets S2 and S3.

Next, we generated DNA libraries, which were subsequently sequenced. The obtained reads were aligned to known Rosaceae microRNAs (miRs) from miRBase, for the detection of putative pear miRs. A miR was called “present” based on the criteria described in methods. In addition, we termed miRs as “tissue-specific” if they had no known presence in other tissues. In the exudates of all the studied genotypes combined, we identified a total number of 28 sequences that are known to be mature miRs in other Rosaceae species. Three mature miRs with single reads were discarded from further analysis. Analysis of the stigma samples revealed a total number of 71 Rosaceae known mature miRs, 22 of which were with single reads, and were thus also discarded from further analysis. Out of the remaining 49 miRs, 24 were stigma specific, while 25 were common to both stigma and exudates (Fig. 2A and supporting dataset S1). All 49 stigma miRs were present in *P. syriaca*, while nine of them were shared by all three pear species. The absence of many stigma miRs from the exudates indicates the selective secretion of miRs from the stigma.

ExRNAs of various sizes can be secreted from cells through several mechanisms, some of which involve RNA-binding proteins that facilitate miR loading to extracellular vesicles (EVs). The EVs can be derived from the trans-Golgi network (TGN) and/or multivesicular body (MVB) endosomes [11]. We therefore examined, using Cryo-TEM, the stigma exudates of *P. syriaca* and identified EVs of various sizes, of diameters ranging between 50 and 800 nm (Figure 1D and supporting dataset S5). To our knowledge, this is the first report of EVs in stigma exudates. Based on this observation, it is possible that the exudate miRs we identified were secreted from the papilla cells via EVs. Nevertheless, the exact cargo of these EVs is yet to be determined.

To further study the possible function of the identified miRs, we used in silico target prediction databases (psRNATarget), and identified in the *Arabidopsis thaliana* genome (which has been well functionally characterized [12]) a total number of 167 target genes (Fig. 2B and supporting dataset S2). The stigma-specific miRs have 120 potential gene targets, while the exudate abundant miRs are predicted to target another 47 genes. Interestingly, for seven pear identified miRs (stigma or exudate), we were not able to identify any targets in Arabidopsis. However, the lack of targets in the model plant is likely due to pear-specific targets, yet to be deciphered within the pear genome. Future functional characterization of the pear genome will allow more accurate identification of miR targets in pear.

Next, to learn more about the functional features of the predicted targets, we performed gene ontology (GO) analysis. Overall, the predicted targets were associated with a wide range of biological processes (BPs). The 120 genes predicted to be targeted by stigma-specific miRs showed enrichment associated with 34 BPs (*P* < 0.005), whereas the 47 genes predicted to be targeted by exudate abundant miRs corresponded to 25 of these target BPs (*P* < 0.005). The nine BPs targeted by stigma-specific miRs only, included metabolic pathways, reproductive pathways, and developmental processes of different tissues (Fig. 2B and supporting dataset S3).

Exploring the Arabidopsis pollen transcriptome [13] revealed 25 pollen transcribed genes predicted to be targeted by the exudate miRs (supporting dataset S4), including HA8, recently reported to play a crucial role in pollen germination and pollen tube growth [14]. These findings indicate that the exudate residing miRs are potentially involved in transcriptional regulation of the landing pollen.

In terms of future prospects, recent studies have shown that plant extracellular small RNAs or miRs can be transmitted to various microorganisms and interfere with their transcriptional regulation [15, 16]. Therefore, mining genomes of stigma interacting organisms/microorganisms will potentially identify additional targets for the miRs reported here. Our findings thus open new research avenues and suggest novel RNA tools for improving *Pyrus* crop protection against several pathogens and diseases [17, 18].

## Supporting information

Full details of the materials and methods used here and supporting dataset S5 are provided in supplementary information.

## Supplementary Material

pgad332_Supplementary_Data

## Data Availability

Raw data and supporting datasets (#1–#4) were submitted to NCBI GEO (Gene Expression Omnibus) repository. This data can be accessed through: https://www.ncbi.nlm.nih.gov/geo/query/acc.cgi?acc=GSE225720 using the GEO entry: knqpaouulzoxrwp.
