# Supplementary material for: Discovery of microRNAs in Pyrus stigma exudates opens new research avenues in Horticulture
Source: PNAS Nexus. 2023 Oct 12;2(11):pgad332. doi: 10.1093/pnasnexus/pgad332 (PMC10634471; doi:10.1093/pnasnexus/pgad332)
Supplement: pgad332_Supplementary_Data [file pgad332_supplementary_data.docx]

**Supplementary Information**

- Supporting dataset 5
- Extended materials and methods.
- References.


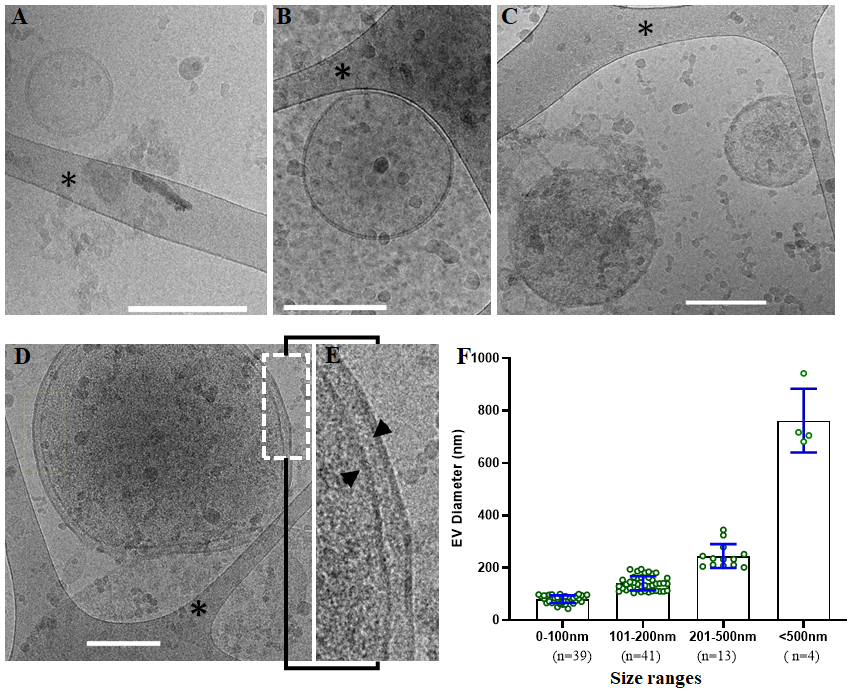


**Supporting dataset 5** - **Cryo-TEM imaging of stigma exudate extracellular vesicles.**

Extracellular vesicles (EV) were isolated from *Pyrus syriaca* exudate and analyzed by Cryo-TEM as described in the methods*.* **A-D)** Shown are representative micrographs of EVs of various sizes. Asterisks indicate the grid’s perforated carbon film. **E)** Higher magnification image of the white boxed area in E. Black arrow points to the double leaflets of the EV’s membrane. **F)** Histogram presenting the heterogeneity in the EV's diameter. The total observed EVs were segregated into columns representing four size ranges. (n=97, ±SE). Scale bars **(A-D)** =200nm.

**Extended materials and methods**

**Plant materials**

The global fruit production of *Pyrus* is challenged by stigma associated obstacles. This includes self-incompatibility [1] and the pathogenic bacteria *Erwinia amylovora*, which colonizes the stigma during fire blight's floral epiphytic phase [2]. Nevertheless, some Israeli farmers and horticulturists suspect that *Pyrus syriaca* which is native to the upper Galilee region of northern Israel (and other parts of the eastern Mediterranean) may harbor resilient traits against the indicated challenges. Therefore, we conducted a comparative study in which the stigma biology was investigated in *Pyrus syriaca* and other cultivated *Pyrus* species, which are grown commercially in northern Israel. The following species were studied: *Pyrus communis* and *Pyrus pyrifolia. Pyrus communis* (European pear, cv. “Spadona”) *and Pyrus pyrifolia* (Asian pear, cv. “Hosui” and “Shinsui”) were grown in commercially cultivated orchards which are located at Kibbutz Yiftah, in the upper Galilee region of northern Israel (Altitude 430 meters). In addition, three wild populations of *Pyrus syriaca* were studied in the following sites of northern Israel: Manara (Altitude 450 meters), SASA, (Altitude 830 meters) and Golan (Altitude 900 meters). Blooming branches were collected at the field during spring 2018 (March-April), immediately placed in water cans and transferred after removing all the open flowers, for overnight acclimation in growth room (day/night cycles of 16 h at 24 °C and 8 h at 18 °C, humidity of 40–60% and white light of 120–130 µmol/m2 sec).

**Stigma viability assays**

For stigma viability assays, newly opened flowers were sampled at 12 hours after anthesis (HAA) or 72 HAA. Nine stigma lobes were then randomly excised, from four to six flowers that were used for each time point. The viability of the stigmatic cells was determined by simultaneous application of the live/dead stains Fluorescein diacetate (FDA, Sigma) and Propidium Iodide (PI, Sigma), as reported earlier. [3] The FDA and PI fluorescent signals were detected by laser scanning confocal microscope - LSM710 (Zeiss) with a Plan-Apochromat 20 ×0.8 numerical aperture air objective lens, using the setup conditions mentioned previously. [4] Quantification of dead cells was performed on confocal micrographs using the image J software, as described earlier, with slight modification of using image J color deconvolution plugin (<https://imagej.net/plugins/colour-deconvolution>).

**Exudate collection and RNA extraction**

The stigma exudate was collected in a nondestructive manner as described earlier, [5] using slight modifications. In brief, after removal of the anthers from 12 HAA flowers, the stigma of 50 flowers were rinsed several times with 200µl of double distilled water and collected in 1.5-mL Eppendorf. Due to the exudate’s low volume, 500-600 flowers of each genotype were pooled together for each sample. Potential contamination (pollen, debris or microorganisms) was then removed by centrifugation (15000g, 10 min). For isolation of small RNAs, we used Silicon-Carbide matrix based RNA purification kit (cf-RNA isolation kit, NORGEN BIOTEK, #29600) which yield high quality and purity total RNA, including small RNAs from cell free systems.[6] Equal volume of Lysis Buffer-A from the kit was mixed with the cleaned exudate to prevent any degradation, frozen in liquid nitrogen and stored in -80⁰C. After collecting the exudate and stigma from all the three genotypes, total RNA was extracted using the manufacturer’s protocol. The extracted RNA was eluted in 50µl ultra-pure water, its quality was electrophoretically assessed by Agilent 2100 Bioanalyzer [7], then frozen in Liquid nitrogen and stored in -80⁰C.

**Library preparations and Sequencing**

Libraries were prepared using CATS Small RNA kit (C05010044), which allow generation of DNA libraries from picogram amounts of RNA [8]. Library quantity and pooling were measured by Qubit (dsDNA HS, Q32854). The pool was size selected by 4% agarose gel. Library quality was measured by Tape Station (HS, 5067-5584). Libraries were sequenced by NextSeq 500 high output kit V2, 75 cycle single-end (Illumina, 20024906) using a NextSeq 500 machine (Illumina) with ~8 million reads per sample. Sample denaturation and loading was according to manufacturer instruction (Illumina).

**miRNA identification**

Raw reads were processed to remove technical and low-quality bases according to the CATS kit recommendations. Cutadapt, v1.1 (<http://cutadapt.readthedocs.org/en/stable/>) [9], was used with a quality threshold of 32, removing adapter and poly-A sequences, that were added by the CATS kit, and keeping reads 18 to 28 bases long. Reads with an overall low quality were filtered out using fastq_quality_filter (which is part of the FASTX package, v0.0.14, <http://hannonlab.cshl.edu/fastx_toolkit/>) with parameters –q20 (quality threshold) and –p 85 (percentage). Processed reads were then analyzed with the miRExpress pipeline, v2.0, [10] using the ‘Raw_data_parse’, ‘alignmentSIMD’ and ‘analysis’ steps. Reference microRNAs were taken from miRBase, version 22, (<https://www.mirbase.org/>) [11], using all *Rosaceae* species in the database - *Fragaria vesca*, fve; *Malus domestica*, mdm; and *Prunus persica*, ppe. The reference included 606 precursor microRNAs and 667 mature microRNAs. Alignment allowed up to 7% mismatches and mature microRNA assignment allowed for offset of up to 11 bases and required at least 50% overlap. Some mature microRNAs in this dataset shared the exact same sequence, either being the same mature miR coming from several precursors, or due to the gathering of miRs from several species. Hence, a unique representative was kept for each mature miR sequence, averaging the counts from all its appearances in the dataset. This unique mature miRs dataset included 355 mature miRs. The table of raw counts for each miR in each sample was used to define “presence” or “absence” for each mature miR, at the sample, tissue and species levels. A miR was termed “present” in a sample if at least 2 reads were assigned to it in this sample. For both tissue and species levels, a miR was defined as “present” if it was present in any of the samples of that tissue or species, respectively. A further definition of “tissue-specific” was made for miRs that were present in one tissue and had no evidence in the other tissue. Mature miRs that were present in one tissue and the maximum evidence in the other tissue was a single read, were therefore neither common to both tissues nor tissue-specific and were discarded from further analysis.

**miRNA targets prediction**

Targets were predicted by submitting the identified miRs to “psRNATarget” - a small RNA target analysis server (<https://www.zhaolab.org/psRNATarget/analysis>).[12] The cDNA library of *Arabidopsis thaliana* from TAIR version10 was selected for target search (with subtraction of miRNA genes). All the parameters were set to the default value, except “expectation” which was set to ≤2.5

**GO enrichment and KEGG pathway analyses**

The gene ontology (GO) enrichment analysis of the target genes in Arabidopsis was performed using AgriGO2 server (<http://systemsbiology.cau.edu.cn/agriGOv2/>).[13] All the GO terms with a FDR (false discovery rate) < 0.05 were selected, and redundant terms were collapsed using REVIGO (<http://revigo.irb.hr/>).[14] The visualization of GO enrichment through dot plot was generated using ggplot2 package ([https://cran.r-project.org/web/packages/ggplot2](https://cran.r-project.org/web/packages/ggplot2/index.html)

**Cryo-TEM**

Extracellular vesicles were isolated as described earlier. In brief, stigma exudates were collected from *Pyrus syriaca* as mentioned above, then concentrated by ultracentrifuge (Optima XE 90K, Beckman Coulter) for 1 hour at 180,000g, followed by pellet resuspension in 30 µl of PBS buffer, which was cooled on ice.[15] Cryo-TEM specimens were prepared in a controlled environment vitrification system as described previously. In brief, three µl droplet of the concentrated exudate were loaded onto a grid which was swiftly plunged into liquid ethane at its freezing point (-183 C) and stored in liquid nitrogen. Specimens were then moved into an Oxford CT-3500 cryo-holder (Philips TEM) and equilibrated below -175 C. Cryogenic transmission electron microscopy (cryo-TEM) imaging was then performed by a Phillips CM120 microscope, operated at 120 kV.[16]

**Statistical analysis and software**

The number of viable papilla cells (presented in Figure 1B) with strong FDA fluorescence, and the number of dead papilla cells showing red PI fluorescence (from the nucleus) were counted using Image J ([https://imagej.net/software/Fiji/](https://eur05.safelinks.protection.outlook.com/?url=https%3A%2F%2Fimagej.net%2Fsoftware%2FFiji%2F&data=05%7C01%7Cyoril%40migal.org.il%7C91ce2c822f274c1ef70008dbc0b66fd6%7Ca8046269518d4482ba873301c601447a%7C0%7C0%7C638315662568918855%7CUnknown%7CTWFpbGZsb3d8eyJWIjoiMC4wLjAwMDAiLCJQIjoiV2luMzIiLCJBTiI6Ik1haWwiLCJXVCI6Mn0%3D%7C3000%7C%7C%7C&sdata=EcjZ%2FVcuWxo2Shx9osKBkKpWZwjJ%2FS2V%2FhoNVVlqecg%3D&reserved=0)). The percentage of viable and dead papilla cells per stigma was calculated (n=9). Significant differences between the viable and non-viable papilla cells at 12h and 72h post-anthesis were confirmed by applying the Mann–Whitney nonparametric test using GraphPad 10.0.3.

**Data availability**

The raw datasets of the RNA sequencing presented in this study were submitted to NCBI GEO (Gene Expression Omnibus) repository. This data can be accessed through <https://www.ncbi.nlm.nih.gov/geo/query/acc.cgi?acc=GSE225720>

using the GEO entry knqpaouulzoxrwp.

**Methods References**

[1] H. Claessen, W. Keulemans, B. Van de Poel, and N. De Storme, “Finding a compatible partner: Self-incompatibility in european pear (Pyrus communis); molecular control, genetic determination, and impact on fertilization and fruit set,” *Front. Plant Sci.*, vol. 10, no. April, p. Article 407, 2019, doi: 10.3389/fpls.2019.00407.

[2] Q. Zeng, J. Puławska, and J. Schachterle, “Early events in fire blight infection and pathogenesis of Erwinia amylovora,” *J. Plant Pathol.*, vol. 103, pp. 13–24, 2021, doi: 10.1007/s42161-020-00675-3.

[3] Z. Gao *et al.*, “KIRA1 and ORESARA1 terminate flower receptivity by promoting cell death in the stigma of Arabidopsis,” *Nat. Plants*, vol. 4, no. 6, pp. 365–375, 2018, doi: 10.1038/s41477-018-0160-7.

[4] V. Ambastha, Y. Friedmann, and Y. Leshem, “Laterals take it better – Emerging and young lateral roots survive lethal salinity longer than the primary root in Arabidopsis,” *Sci. Rep.*, vol. 10, no. 1, pp. 1–11, 2020, doi: 10.1038/s41598-020-60163-7.

[5] P. L. Pusey, D. R. Rudell, E. A. Curry, and J. P. Mattheis, “Characterization of stigma exudates in aqueous extracts from apple and pear flowers,” *HortScience*, vol. 43, no. 5, pp. 1471–1478, 2008, doi: 10.21273/hortsci.43.5.1471.

[6] A. Yeri *et al.*, “Total extracellular small RNA profiles from plasma, saliva, and urine of healthy subjects,” *Sci. Rep.*, vol. 7, no. October 2016, pp. 1–13, 2017, doi: 10.1038/srep44061.

[7] A. Schroeder *et al.*, “The RIN: An RNA integrity number for assigning integrity values to RNA measurements,” *BMC Mol. Biol.*, vol. 7, pp. 1–14, 2006, doi: 10.1186/1471-2199-7-3.

[8] A. Turchinovich, H. Surowy, A. Serva, M. Zapatka, P. Lichter, and B. Burwinkel, “Capture and Amplification by Tailing and Switching (CATS), Anultrasensitive ligation-independent method for generation of DNA libraries for deep sequencing from picogram amounts of DNA and RNA,” *RNA Biol.*, vol. 11, no. 7, pp. 817–828, 2014, doi: 10.4161/rna.29304.

[9] Marcel Martin, “Cutadapt removes adapter sequences from high-throughput sequencing reads.,” *EMBnet.journal*, vol. 17, no. 1, pp. 10–12, 2011.

[10] W. C. Wang, F. M. Lin, W. C. Chang, K. Y. Lin, H. Da Huang, and N. S. Lin, “MiRExpress: Analyzing high-throughput sequencing data for profiling microRNA expression,” *BMC Bioinformatics*, vol. 10, p. 328, 2009, doi: 10.1186/1471-2105-10-328.

[11] A. Kozomara, M. Birgaoanu, and S. Griffiths-Jones, “MiRBase: From microRNA sequences to function,” *Nucleic Acids Res.*, vol. 47, no. Database issue, pp. D155–D162, 2019, doi: 10.1093/nar/gky1141.

[12] X. Dai, Z. Zhuang, and P. X. Zhao, “PsRNATarget: A plant small RNA target analysis server (2017 release),” *Nucleic Acids Res.*, vol. 46, no. W1, pp. W49–W54, 2018, doi: 10.1093/nar/gky316.

[13] T. Tian *et al.*, “AgriGO v2.0: A GO analysis toolkit for the agricultural community, 2017 update,” *Nucleic Acids Res.*, vol. 45, no. W1, pp. W122–W129, 2017, doi: 10.1093/nar/gkx382.

[14] F. Supek, M. Bošnjak, N. Škunca, and T. Šmuc, “Revigo summarizes and visualizes long lists of gene ontology terms,” *PLoS One*, vol. 6, no. 7, 2011, doi: 10.1371/journal.pone.0021800.

[15] M. Y. Konoshenko, E. A. Lekchnov, A. V. Vlassov, and P. P. Laktionov, “Isolation of Extracellular Vesicles: General Methodologies and Latest Trends,” *Biomed Res. Int.*, vol. 2018, no. 8545347, 2018, doi: 10.1155/2018/8545347.

[16] N. Koifman, I. Biran, A. Aharon, B. Brenner, and Y. Talmon, “A direct-imaging cryo-EM study of shedding extracellular vesicles from leukemic monocytes,” *J. Struct. Biol.*, vol. 198, no. 3, pp. 177–185, 2017, doi: 10.1016/j.jsb.2017.02.004.
